# Supplementary material for: Tonotopic and non-auditory organization of the mouse dorsal inferior colliculus revealed by two-photon imaging
Source: eLife. 2019 Oct 15;8:e49091. doi: 10.7554/eLife.49091 (PMC6834370; doi:10.7554/eLife.49091)
Supplement: Transparent reporting form [file elife-49091-transrepform.pdf]

eLife's transparent reporting form

We encourage authors to provide detailed information *within their submission* to facilitate the interpretation and replication of experiments. Authors can upload supporting documentation to indicate the use of appropriate reporting guidelines for health-related research (see EQUATOR Network), life science research (see the BioSharing Information Resource), or the ARRIVE guidelines for reporting work involving animal research. Where applicable, authors should refer to any relevant reporting standards documents in this form.

If you have any questions, please consult our Journal Policies and/or contact us: editorial@elifesciences.org.

Sample-size estimation

- You should state whether an appropriate sample size was computed when the study was being designed
- You should state the statistical method of sample size computation and any required assumptions
- If no explicit power analysis was used, you should describe how you decided what sample (replicate) size (number) to use

Please outline where this information can be found within the submission (e.g., sections or figure legends), or explain why this information doesn't apply to your submission:

No explicit power analysis could be performed beforehand because of insufficient prior measurements. The spatial distribution was first observed in a subset of the animals. Subsequently we increased the number of animals to confirm these findings.

Replicates

- You should report how often each experiment was performed
- You should include a definition of biological versus technical replication
- The data obtained should be provided and sufficient information should be provided to indicate the number of independent biological and/or technical replicates
- If you encountered any outliers, you should describe how these were handled
- Criteria for exclusion/inclusion of data should be clearly stated
- High-throughput sequence data should be uploaded before submission, with a private link for reviewers provided (these are available from both GEO and ArrayExpress)

Please outline where this information can be found within the submission (e.g., sections or figure legends), or explain why this information doesn't apply to your submission:

The number of animals used for each data set is stated under “Animals” in “Materials and Methods”, or in case of animals aligned for spatial distribution in “Results” under “Spatial distribution of frequency tuning”.
The total number of cells analyzed for each experiment is stated in the “Results” section; for marker expression count, the number of cells is stated in the paragraph “Expression of GCaMP6s in IC subpopulation with transgenic mice”; for two photon imaging, under “Sound-evoked changes in FGCaMP of IC neurons”; for ground truth recordings, under “Electrophysiological correlates of response classes”.

Statistical reporting

- Statistical analysis methods should be described and justified
- Raw data should be presented in figures whenever informative to do so (typically when N per group is less than 10)
- For each experiment, you should identify the statistical tests used, exact values of N, definitions of center, methods of multiple test correction, and dispersion and precision measures (e.g., mean, median, SD, SEM, confidence intervals; and, for the major substantive results, a measure of effect size (e.g., Pearson's r, Cohen's d)
- Report exact p-values wherever possible alongside the summary statistics and 95% confidence intervals. These should be reported for all key questions and not only when the p-value is less than 0.05.

Please outline where this information can be found within the submission (e.g., sections or figure legends), or explain why this information doesn’t apply to your submission:

Whenever possible, raw data points are presented as beeswarm plots or scatter plots (i.e. Figures 4C, 5E-F, 6A, Figure 3 – figure suppl 1). In cases where the display of individual data points became confusing (e.g. due to many repeated values), a heatmap (Figure 6C) or histograms (Figure 6B, 7, 8C-D, Figure 2 – figure suppl 1G) are presented.

The error bars in Figures 1M, Figure 2 – figure suppl 1G & Figure 3 – figure suppl 1C-E were defined in their respective legends, as well as the N. In Figure 6D-E, the N for each data point is presented as the size of the symbol with corresponding look up scale as an inset. All indications of dispersion by “±” signs in the text are accompanied by an indication of the precision measure used (e.g., “average ± s.d.: 77 ± 13%”).

Statistical tests for difference in cell size (Figure 4) are provided in the “Results” section under “Relationship between cell type and soma size”. Statistics for determining significant response from frequency response areas (Figures 3 & 5) are described in the “Material and Methods” section under the headings “Analysis of Frequency Response Areas and Response Classification”.

(For large datasets, or papers with a very large number of statistical tests, you may upload a single table file with tests, Ns, etc., with reference to sections in the manuscript.)

Group allocation

- Indicate how samples were allocated into experimental groups (in the case of clinical studies, please specify allocation to treatment method); if randomization was used, please also state if restricted randomization was applied

eLife Sciences Publications, Ltd is a limited liability non-profit non-stock corporation incorporated in the State of Delaware, USA, with company number 5030732, and is registered in the UK with company number FC030576 and branch number BR015634 at the address 1st Floor, 24 Hills Road, Cambridge CB2 1JP | August 2014

- Indicate if masking was used during group allocation, data collection and/or data analysis

Please outline where this information can be found within the submission (e.g., sections or figure legends), or explain why this information doesn't apply to your submission:

Group allocation does not apply to our project, as there was no comparison between control and experimental groups.

Due to practicalities, the experimenter (ABW) was not blinded to the genotype of the animals at the time of experiment nor at the time of analysis. However, the classification of response class, calculation of frequency response area and the fitting of response kinetics were performed with the same, semi-automatic algorithm with minimal manual curation to ensure that all analyzed regions-of-interest were treated in the same way.

Additional data files ("source data")

- We encourage you to upload relevant additional data files, such as numerical data that are represented as a graph in a figure, or as a summary table
- Where provided, these should be in the most useful format, and they can be uploaded as "Source data" files linked to a main figure or table
- Include model definition files including the full list of parameters used
- Include code used for data analysis (e.g., R, MatLab)
- Avoid stating that data files are "available upon request"

Please indicate the figures or tables for which source data files have been provided:

Figure 1, Figure 1 – figure supplement 1: counting data
Figure 3 –figure supplement 1, Figure 4, Figure6, Figure 7 and Figure 8: Fluorescence kinetics, CF, location of cells, genotype.
Code for fitting spatial distribution in Igor Pro.
Figure 5: model parameters for fitting ground-truth data.

Raw imaging data is very large in size, exceeding the available space allocated for uploading.
